# Supplementary material for: TGF-β-Upregulated Lnc-Nr6a1 Acts as a Reservoir of miR-181 and Mediates Assembly of a Glycolytic Complex
Source: Noncoding RNA. 2022 Sep 15;8(5):62. doi: 10.3390/ncrna8050062 (PMC9498520; doi:10.3390/ncrna8050062)
Supplement: Supplementary file 1 [file ncrna-08-00062-s001.zip › Supplementary material/Supplementary Figures.pdf]

SUPPLEMENTARY DATA

Supplementary Figure S1**.** Mapped 3′ and 5′ ends of lnc-pri-miR-181a2/b2.
(**A**) Sequencing results of lnc-pri-miR-181a2/b2 3′RACE products amplified by poly(A) polymerase dependent method. On the left, sequencing of PCR products revealed the boundary between the adaptor primer and lnc-*Nr6a1* sequences. On the right, schematic illustration showing location of used primers (black arrowheads) and 3′ end cleavage sites based on sequence analysis (green arrows). (**B**) On the right, a representative image of PCR product from 5′-RACE procedure; on the left, sequencing of PCR product revealed the boundary between the adaptor primer and *Lnc-Nr6a1* sequence.

Supplementary Figure S2. 3′-end mapping and lnc-*Nr6a1* sequences.
(**A**) and (**B**) Location of mapped 3′-end cleavage products are shown by green arrows on the lnc-pri-miR-181a2/b2. (**C**) Lnc-*Nr6a1-1* isoform cDNA sequence; exons 1, 2 and 3 are colored yellow, blue and gray respectively (**D**) Lnc-pri-miR-181a2/b2 sequence; exons 1, 2 and 3 are colored yellow, blue and gray respectively and mir-181a2 and mi-181b2 in red.

Supplementary Figure S3.
Coding capacity of lnc-*Nr6a1-1* and lnc-*Nr6a1-2* as well as other known coding and noncoding RNAs was predicted by analysis with Coding Potential Calculator software and Coding Potential Assessment Tool.

Supplementary Figure S4. *LNC-NR6A1* human orthologue.
(**A**) Schematic illustration of the genomic location and transcript structure of the *LNC-NR6A1* human orthologue gene. (**B**) Expression of *LNC-NR6A1* orthologue gene in MCF10A human breast epithelial cells and primary human omental mesothelial cells after 24 h of TGF-β treatment. Error bars represent S.D. ****P*<0.001 by two-tailed Student's *t*-test.

Supplementary Figure S5
(**A**) Screenshot of H3K4me3, H3K27ac, ATAC-seq and RNA-seq tracks at *Lnc-Nr6a1* genomic region of NMuMG cells. Position of mir-181a2 and mir-181b2 are shown. (**B**) Scatter plot of expression of miR-181a2-3p and its host gene *LNC-NR6A1* in human cancers. BLCA (Bladder Urothelial Carcinoma), BRCA (Breast invasive carcinoma), COAD (Colon adenocarcinoma), CESC (Cervical squamous cell carcinoma and endocervical adenocarcinoma), PADD (Pancreatic adenocarcinoma), OV (Ovarian serous cystadenocarcinoma), LIHC (Liver hepatocellular carcinoma), HNSC (Head and Neck squamous cell carcinoma) and THCA (Thyroid carcinoma).

Supplementary Figure S6.
Validation by RT-qPCR of expression of genes in *Lnc-Nr6a1*-depleted cells compared to control cells. For each mRNA, results are presented relative to the average value of *Hprt* reference gene. Experiments were performed in triplicate samples. Error bars represent S.D. ****P*<0.001 by two-tailed Student's *t*-test.

Supplementary Figure S7. Cell cycle analysis and cell proliferation.
(**A**) Cell cycle profiles of control cells and *Lnc-Nr6a1*-depleted cells. (**B**) Real-time monitoring of *in vitro* proliferation of *Lnc-Nr6a1*-depleted cells and control cells using the exCELLigence system. The system measures electric impedance across interdigitated microelectrodes integrated on the bottom of tissue culture plates. Error bars represent S.D. (**C**) Rate of proliferation by analysing the slopes of the cell proliferation curves. Error bars represent S.D. ns, not significant by two-tailed Student's *t*-test.

Supplementary Figure S8. Cell cycle analysis and cell proliferation.
(**A**) Cell cycle profiles of control, lnc-*Nr6a1-1*-overexpressing and lnc-*Nr6a1-2*-overexpressing cells. (**B**) Real-time monitoring of *in vitro* proliferation of control, lnc-*Nr6a1-1*-over-expressed and lnc-*Nr6a1-2*-over-expressed cells using the exCELLigence system. Data are from three replicates. Error bars represent S.D. (**C**) Rate of proliferation by analysing the slopes of the cellproliferation curves. Error bars represent S.D. ns, not significant by two-tailed Student's *t*-test.

Supplementary Figure S9. Proliferation and cell adhesion.
(**A**) Real-time monitoring of *in vitro* proliferation of control cells and two independent clones (guide 2 and guide 8) over-expressing the lnc-*Nr6a1* precursor using the exCELLigence system. Data are from three replicates. Error bars represent S.D. (**B**) Rate of proliferation by analysing the slopes of the cell proliferation curves. Error bars represent S.D. ns, not significant by two-tailed Student's *t*-test. (**C**) Substrate cell adhesion assay and (**D**) quantification of crystal violet staining at OD_{590} nm of control and lnc-*Nr6a1-1*-overexpressing cells and clones of guide 2 and guide 8 over-expressing the *Lnc-Nr6a1* gene.

Supplementary Figure S10. Lamin A/C-associated domains.
(A) UCSC Genome Browser overview of lamin A/C ChIP-seq profiles in NMuMG control cells (red) and in *Lnc-Nr6a1*-depleted cells (red) and the corresponding identified LADs. From top to bottom: the complete chromosome 1 (mm9) and representative regions of chromosome 1, chromosome 16 and chromosome 11. (B) Gene ontology analysis of genes associated to LADs partially lost in *Lnc-Nr6a1*-depleted cells. (**C**) Venn diagrams showing the intersection of deregulated genes and LADs associated genes in *Lnc-Nr6a1*-depleted cells. Probability of overlapping based on hypergeometric distribution is provided.

Supplementary Figure S11. Human *LNC-Nr6A1* expression in tumors and normal tissues.
Expression levels of human *Lnc-Nr6a1* gene in different primary tumors versus the corresponding adjacent normal tissues. Expression data were downloaded from the GPIA2 dataset. ACC, adrenocortical carcinoma; BLCA, bladder urothelial carcinoma; CESC, cervical squamous cell carcinoma and endocervical adenocarcinoma; CHOL, cholangio carcinoma; COAD, colon adenocarcinoma; ESCA, esophageal carcinoma; GBM, glioblastoma multiforme; HNSC, head and neck squamous cell carcinoma; LAML, acute myeloid leukemia; LGG, brain lower grade glioma; PAAD, pancreatic adenocarcinoma; PCPG, pheochromocytoma and paraganglioma; SARC, sarcoma; STAD, stomach adenocarcinoma; THCA, thyroid carcinoma; THYM, thymoma; UCEC, uterine corpus endometrial carcinoma; UCS, uterine carcinosarcoma. In brackets are shown the number of primary tumor (T) and adjacent normal tissue (N) samples.

Table S1. Transcriptomic analysis of control and *Lnc-Nr6a1*-depleted NMuMG cells. List contains only genes whose expression (fold change > [1.5]) significantly changed (*P*-adj < 0.05).

Table S2. Gene Ontology enrichment analysis of downregulated genes in *Lnc-Nr6a1*-depleted cells.

Table S3. Transcriptomic profile of control NMuMG cells treated with TGF-β for 2 hours.

Table S4. Transcriptomic profile of *Lnc-Nr6a1*-depleted NMuMG cells treated with TGF-β for 2 hours.

Table S5. The lnc-*Nr6a1-1* protein interactome. Results of iDRiP-mass spectrometry analysis.

Table S6. Genes in LADS lost and LADS gain in *Lnc-Nr6a1*-depleted NMuMG cells

Table S7. Peptide-spectrum match counting for enolase1 coimmunoprecipitation assay

Table S8. Primers and oligonucleotides related to experimental procedures

Movie S1. Wound-healing assay. Time-lapse video microscopy of control, *Lnc-Nr6a1*-depleted, lnc-*Nr6a1-1* overexpressing and lnc-*Nr6a1-2* overexpressing NMuMG cells.

Movie S2. Wound-healing assay. Time-lapse video microscopy of control, lnc-*Nr6a1-1* overexpressing and *Lnc-Nr6a1* geme-upregulated (Guide 2) NMuMG cells.

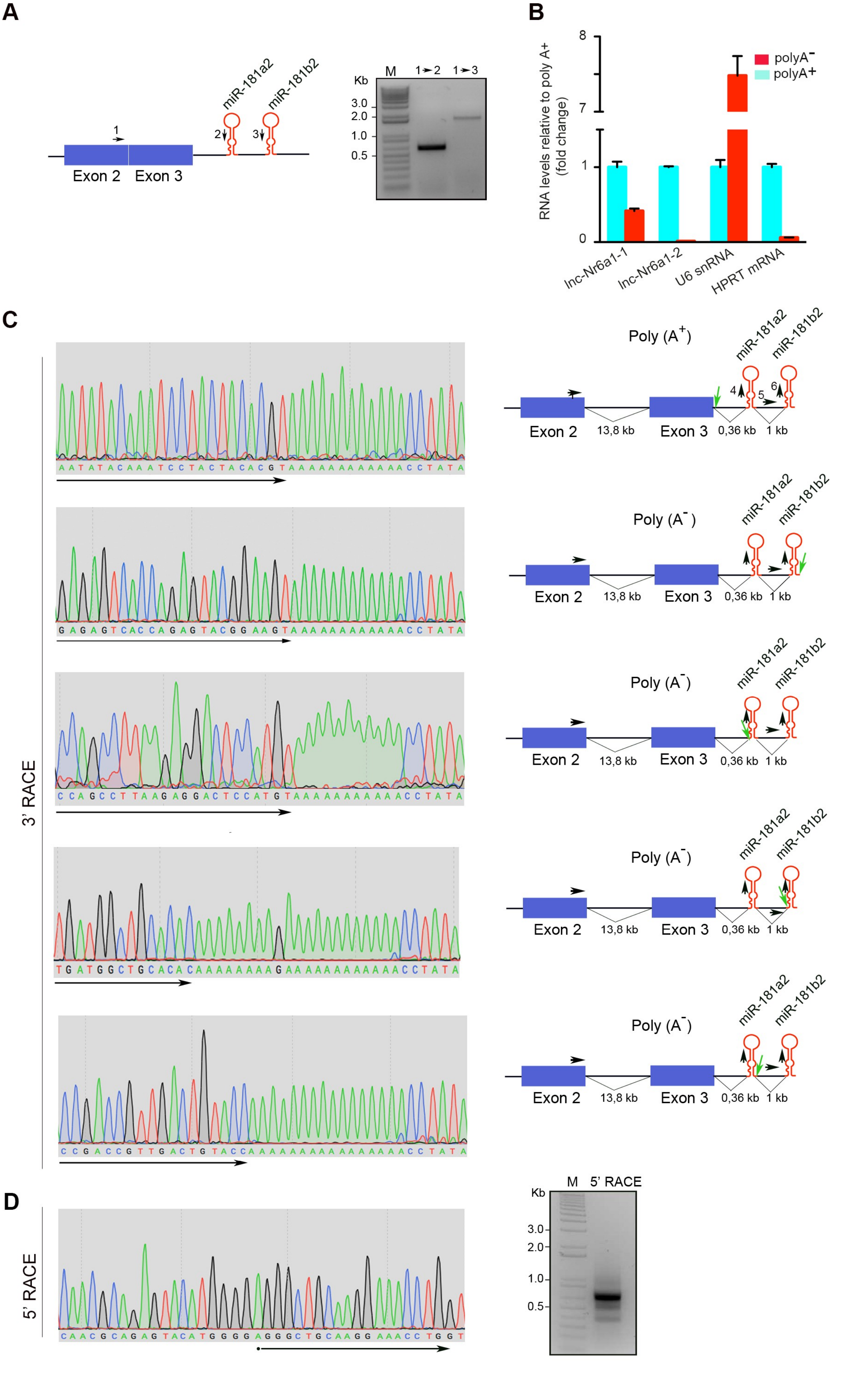

Supplementary Figure S1

A

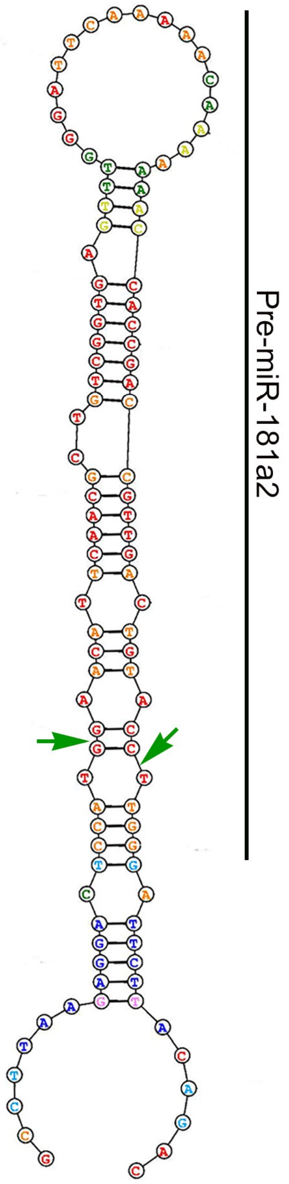

B

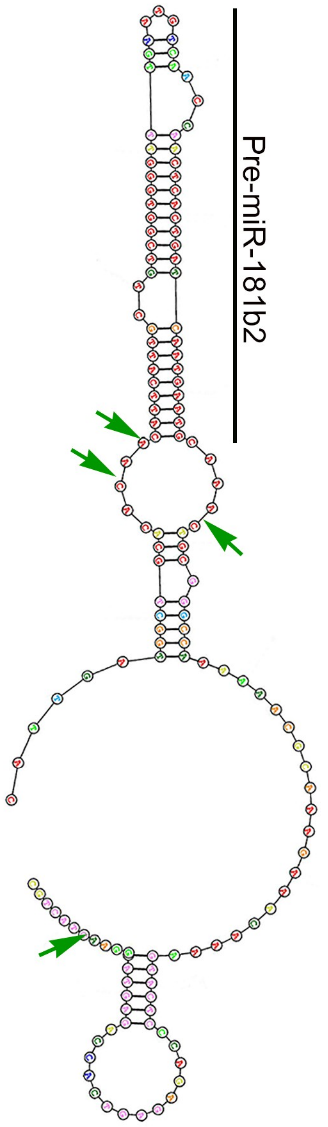

C

Lnc-Nr6a1-1 isoform cDNA sequence

5’agggctgcaaggaaacctggtgcggttcaataccttgtctagcctgggctccaggatcttttttttttttttttttttaaactgacaaac
tcatttttctactgggacaggatgctgtgctggctgaagttccatttctacagcaagaatcctatctggaagcacagaagttgtcctcta
gccacagcagctccaactttttgttgttgctgctttcttctatcacccccttccccttttgacaaagatccaactgtagaaagtcttacgt
aacagttcaggactacttcggttcttttaccggtgaaatgaagctgtgataatgtggtaaatatggggagacaccaagctgtggctctt
gcggacttcagcagcactgaaatgcaacactgtgggctttggcagctgcattccgtactctgaaattcttatatcattactctggcaaac
aagaaggaatttgcttgtttgatttagctctgcagtaaaggttcaaatctgagactggtaaaggaaggagaaagcaacatatccaacc
aatactggaacaatgaactctggtgttcaagttctggctatggaactccgggtaagtctctgtttcttaaggaaaacacggttggaatt
aaatatacaaatcctactacac 3’

D

Lnc-pri-miR-181a2/b2 sequence

5’agggctgcaaggaaacctggtgcggttcaataccttgtctagcctgggctccaggatcttttttttttttttttttttaaactgacaaac
tcatttttctactgggacaggatgctgtgctggctgaagttccatttctacagcaagaatcctatctggaagcacagaagttgtcctcta
gccacagcagctccaactttttgttgttgctgctttcttctatcacccccttccccttttgacaaagatccaactgtagaaagtcttacgt
aacagttcaggactacttcggttcttttaccggtgaaatgaagctgtgataatgtggtaaatatggggagacaccaagctgtggctctt
gcggacttcagcagcactgaaatgcaacactgtgggctttggcagctgcattccgtactctgaaattcttatatcattactctggcaaac
aagaaggaatttgcttgtttgatttagctctgcagtaaaggttcaaatctgagactggtaaaggaaggagaaagcaacatatccaacc
aatactggaacaatgaactctggtgttcaagttctggctatggaactccgggtaagtctctgtttcttaaggaaaacacggttggaatt
aaatatacaaatcctactacacataagtccccagcatgtgttatggtctttcttgtttttgtacattaataattaaagtattactacaaaa
ttgctatattgatgttttcagaatgatagcattgaaaaatatttccacagatatccacttttagtcagcaatgtaaactgcagtctcaca
atcctgcagctcatttctgctttatgcaaactaacttcacagtgtaagtactgttaacttgaaattttgggaagaatgtgagagacccaa
cagcagtggtccttagaatatgagagcaggccaaagatgggcaaccaagccagccttaagaggactccatggaacattcaacgctg
tcggtgagtttgggattcaaaaacaaaaaaaccaccgaccgttgactgtaccttgggattcttacagacaacaagtttcctgaagcaa
aggagtaggctgtacagatatatcatgtgacagacatcacaagatacagtcctgtggcctcttttctaaagggaaccagcaagtttttt
ttttaagcttccatgtatttatattgtctatttttgtaatttcttgataaataagtgtttcattttaacaatttatgggaagtgccatcatag
taccaaatgcactcaaatatcttctagaaatgtgtcattctagtcagaaatagggtgtattaaaaatgtctatttactgtaggaaaaaa
atctagttaagtcttttattctcatttcttctacgtcctttcaaatttttgctttagggtcaagagatttactcagcaaaaatactcacaga
gttactcactgtctatacaaatcatattgtgcctccagtttcagttactgcggttagggatttgacatctccatgagagaaccagataca
tttccatctccatgtgctaatttaaggaattctgggtcatctctgagcctccgtatccaagtatgttttctcaaatgtagaataggagagg
agagaacctcagagttttaatttaatgagaagatatatgagggtactttgaacagtttctggctcacagcaaatgtttactacaaaaat
ttttacttaactgcaatcataatctatactgctttaccttgaaaagggcacttaacgtctcagcttgaaacaaaagatgaagcaatgtct
ctgagggctcctttacagacatcaacatccaagtctcagcccatgtgatcagagctacttacaaggttctcacatagtgccttcttcctc
acaacttttctgtcttgaacaagttcttgctatacagtcaaggtgatctataattatgagtccacaaccatacagatgactagttactaa
catacaacagacttcaaagaaccatagtgggaagaagagccaggagatagaaaagaccaagacaatttcatcaaaaggaaacatt
gatggctgcacacaacattcattgctgtcggtgggtttgaatgtcaaccaactcactgatcaatgaatgcaaactgcgggccaaaaaa
cgcaaagaaacaaaagtactcccagagagtcaccagagtacggaagt

Supplementary Figure S2

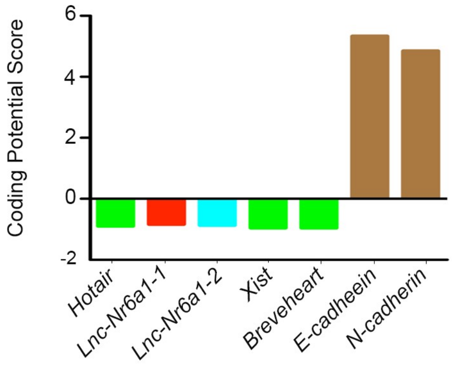

Supplementary Figure S3

A

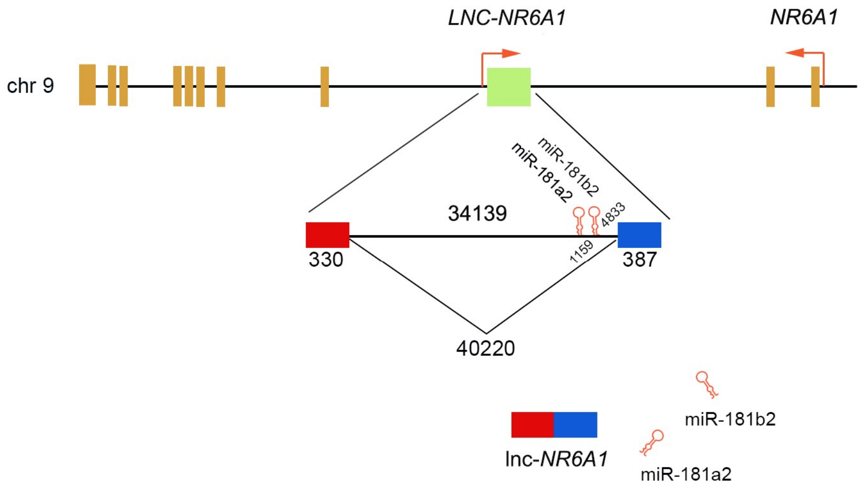

B

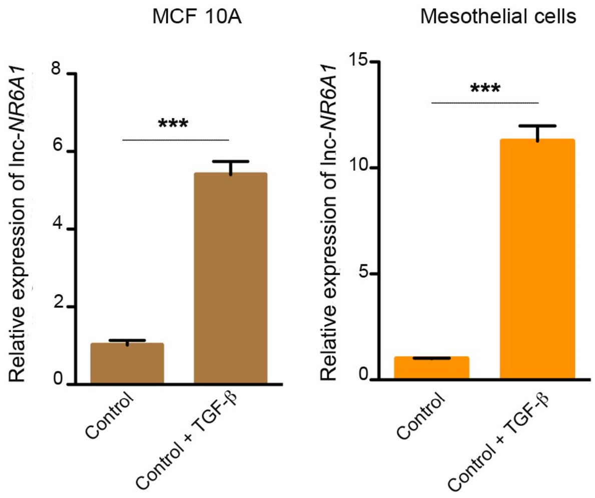

Supplementary Figure S4

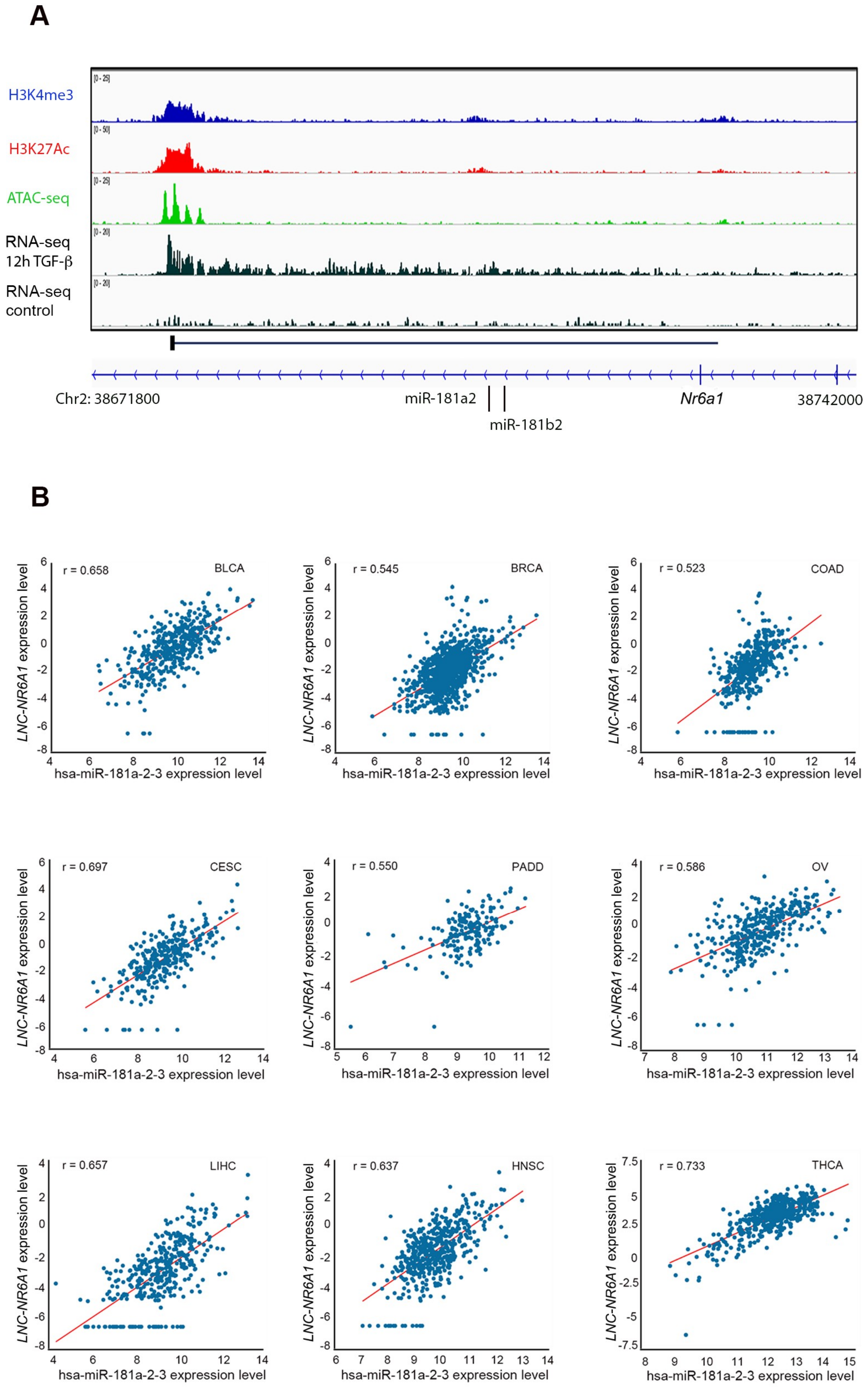

Supplementary Figure S5

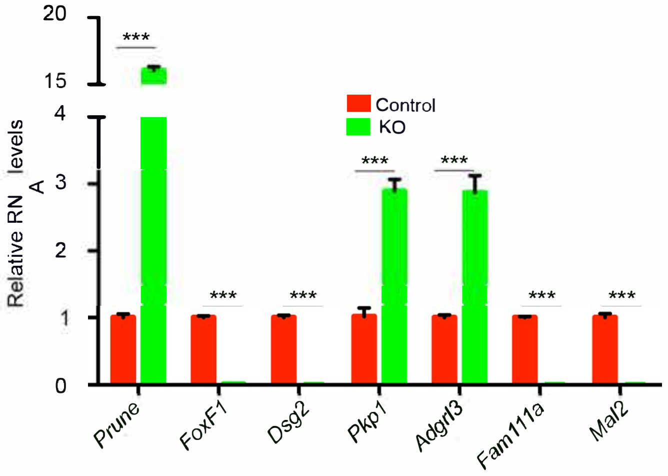

Supplementary Figure S6

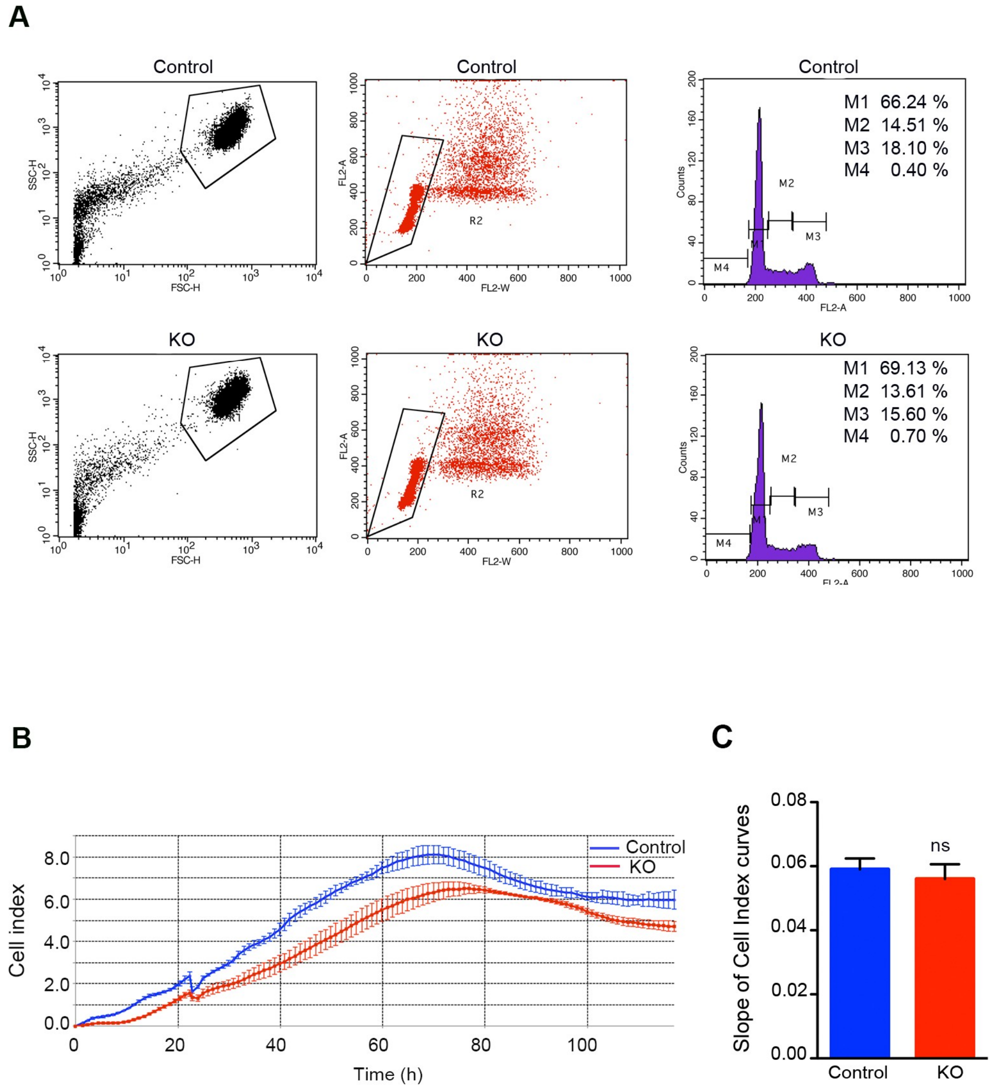

Supplementary Figure S7

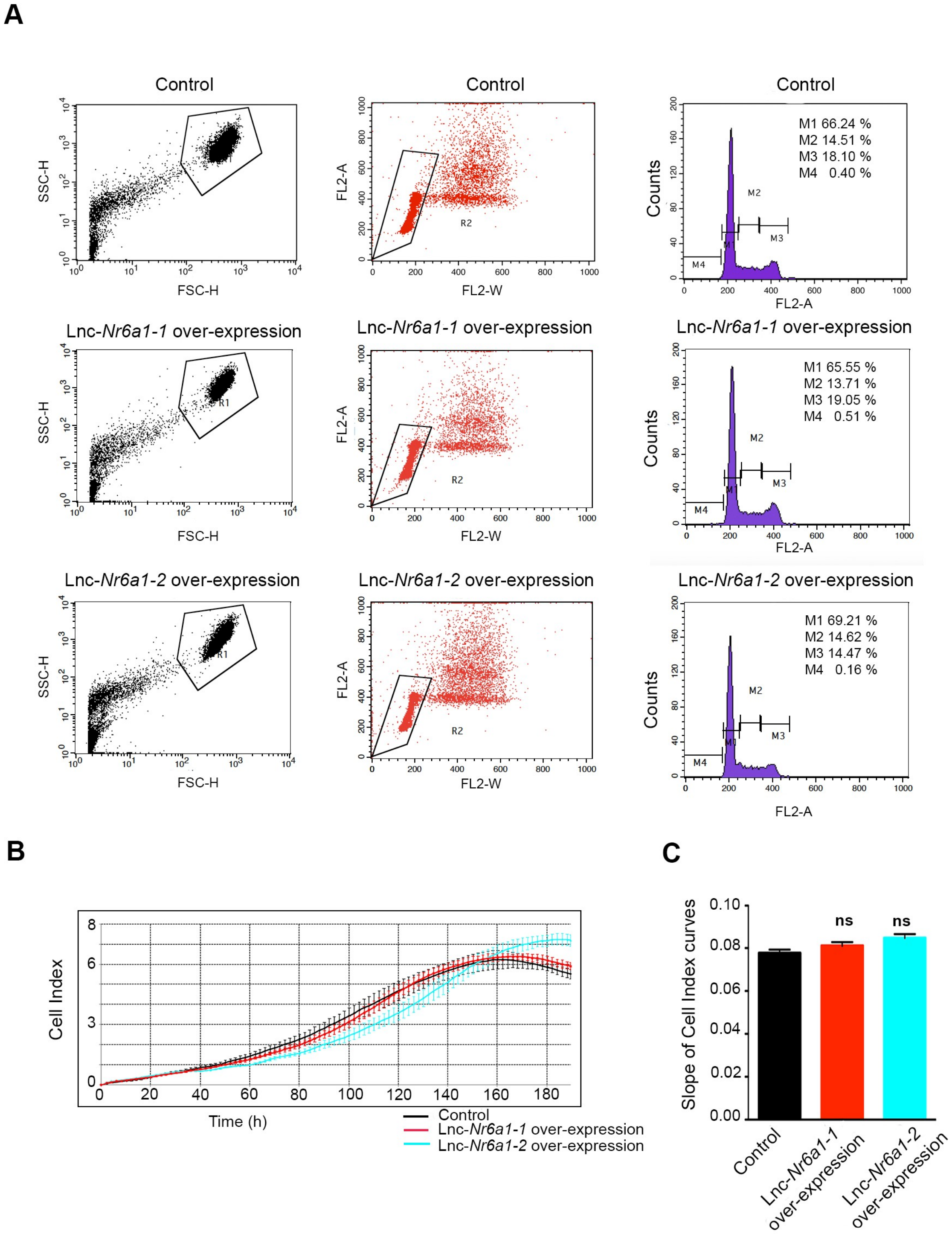

Supplementary Figure S8

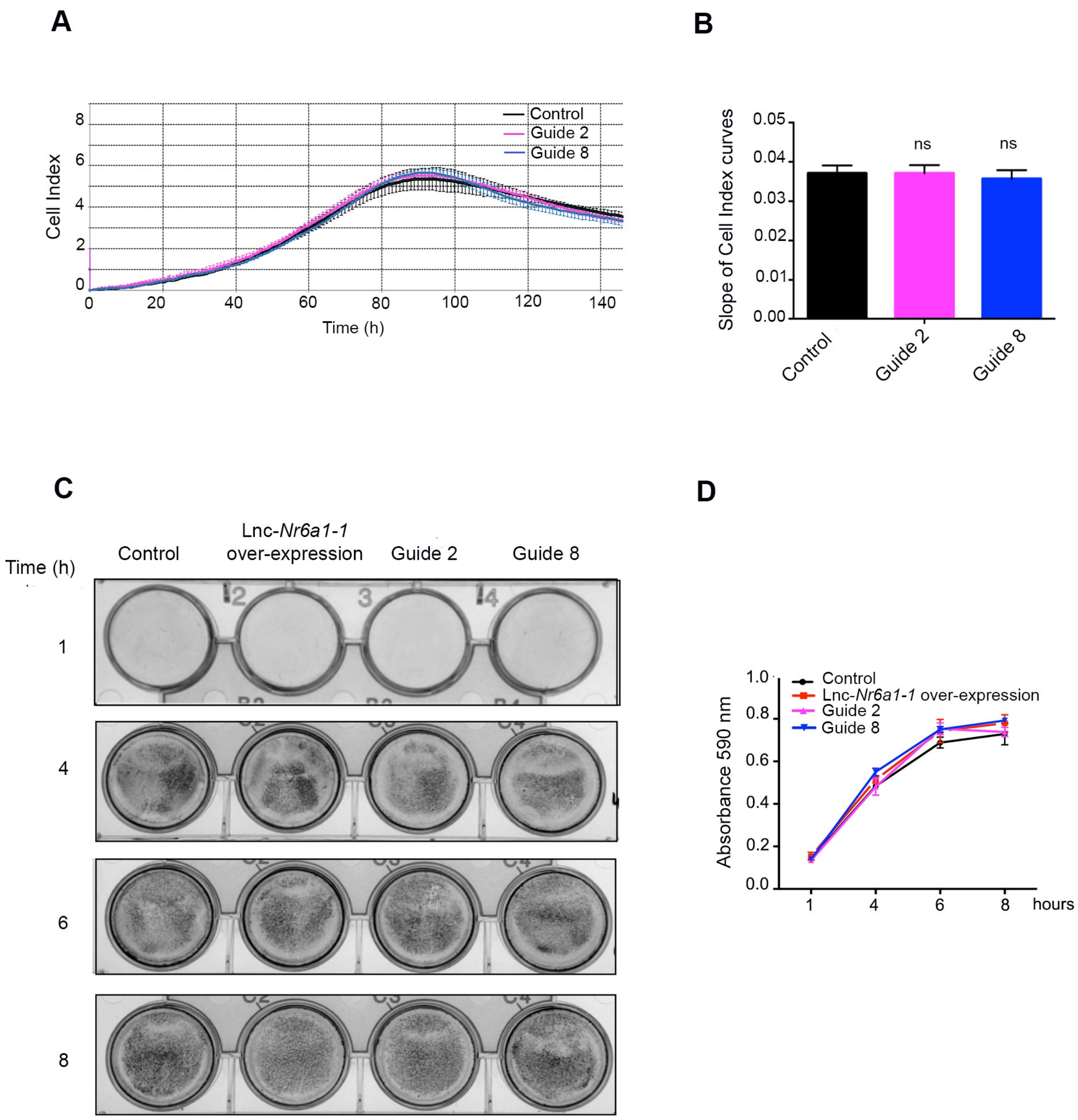

Supplementary Figure S9

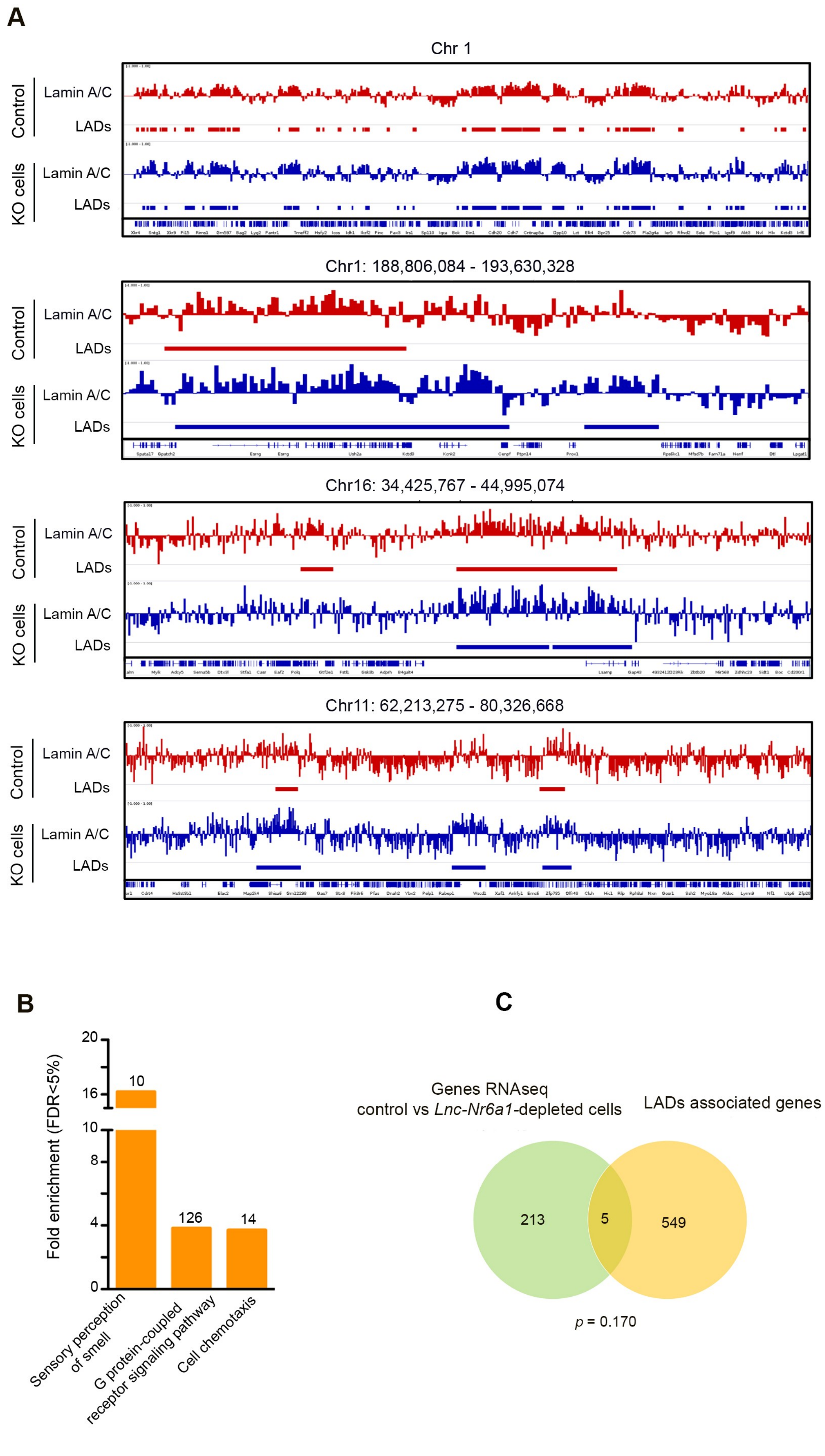

Supplementary Figure S10

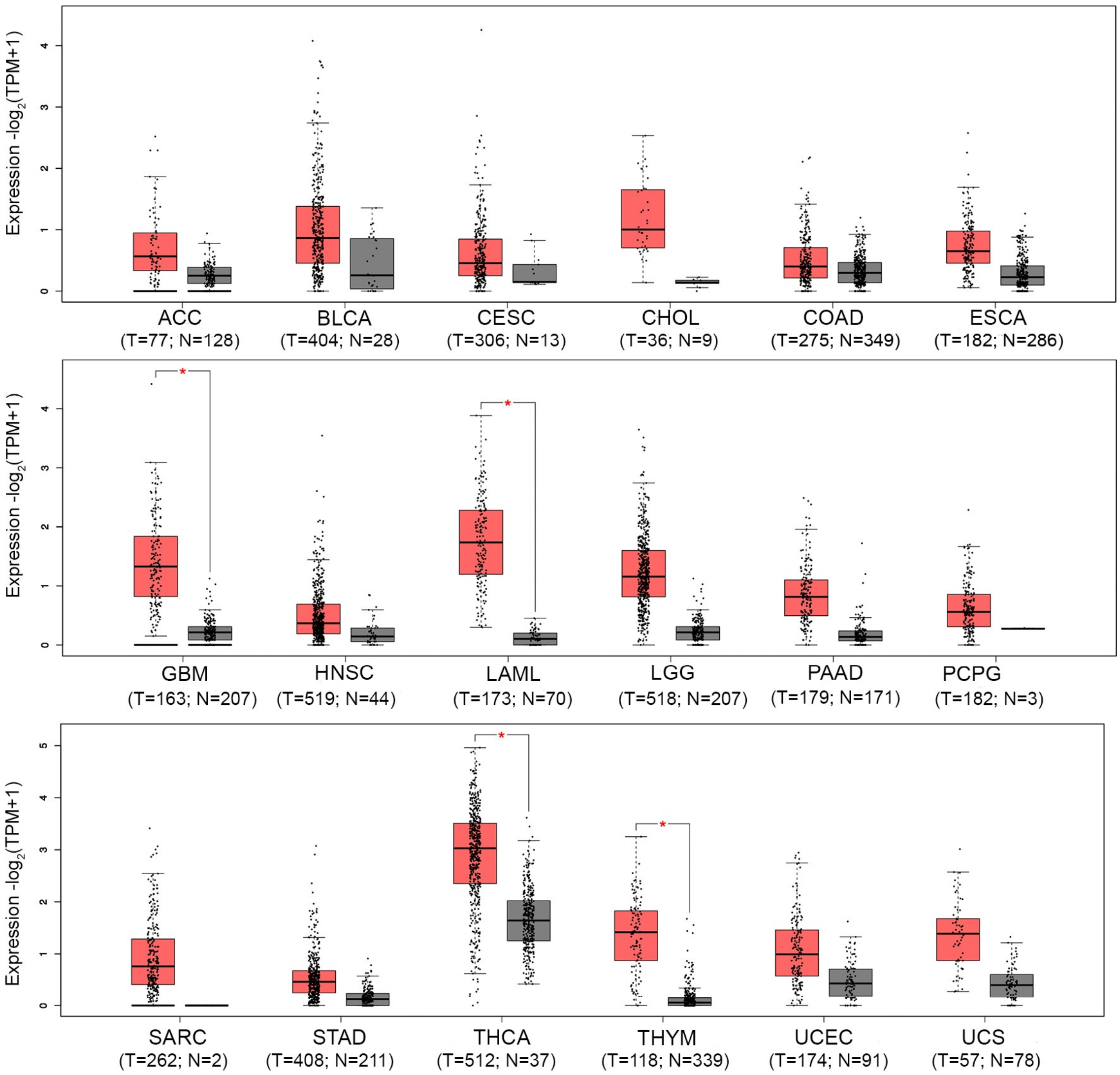

Supplementary Figure S11
